# An 11-week school-based “health education through football” programme improves musculoskeletal variables in 10–12-yr-old Danish school children

**DOI:** 10.1016/j.bonr.2023.101681

**Published:** 2023-04-29

**Authors:** Malte Nejst Larsen, Alessia Terracciano, Trine Kjeldgaard Møller, Charlotte Sandager Aggestrup, Pasqualina Buono, Peter Krustrup, Carlo Castagna

**Affiliations:** a Department of Sports Science and Clinical Biomechanics, SDU Sport and Health Sciences Cluster (SHSC), University of Southern Denmark, Odense, Denmark; b Department of Human Movement Sciences and Wellbeing DISMeB, Parthenope University, Naples, Italy; c Department of Sport and Health Sciences, College of Life and Environmental Sciences, University of Exeter, Exeter, United Kingdom; d Department of Physical Education and Sports Training, Shanghai University of Sport, Shanghai (SUS), China; e Danish Institute for Advanced Study (DIAS), Faculty of Health Sciences, University of Southern Denmark; f Fitness Training and Biomechanics Laboratory, Italian Football Federation, Technical Department, Coverciano (Florence), Italy; g Department of Biomolecular Sciences, School of Exercise and Health Sciences, Carlo Bo Urbino University, Urbino, Italy; h CEINGE-biotecnologie avanzate “Franco Salvatore” s.r.l., Napoli, Italy

**Keywords:** Physical activity, Bone mineral density, Bone health, Soccer, Football program, Children

## Abstract

**Purpose:**

To investigate the effects of the “11 for Health” programme on musculoskeletal fitness.

**Methods:**

A total of 108 Danish children aged 10–12 years participated in the study, with 61 children in the intervention group (IG, 25 girls and 36 boys) and 47 children in the control group (CG, 21 girls and 26 boys). Measurements were conducted before and after an 11-week intervention consisting of twice-weekly 45-min football training sessions for IG or continuation of normal Physical Education program for CG. Whole-body dual X-ray absorptiometry was conducted for evaluation of leg and total bone mineral density as well as bone, muscle and fat mass. Standing Long Jump and Stork balance tests were employed to assess musculoskeletal fitness and postural balance.

**Results:**

During the 11-week study period, leg bone mineral density as well as leg lean body mass increased more (*p* < 0.05) in the intervention group (IG) compared to the control group (CG) (0.021 ± 0.019 *vs* 0.014 ± 0.018 g/cm^2^ and 0.51 ± 0.46 *vs* 0.32 ± 0.35 kg, respectively). Moreover, body fat percentage decreased more in IG than in CG (−0.6 ± 0.1 *vs* 0.1 ± 0.1 %-points, *p* < 0.05). No significant between-group differences were found in bone mineral content. Stork balance test performance increased more in IG than in CG (0.5 ± 2.6 *vs* −1.5 ± 4.4 s, p < 0.05), whereas no between-group differences were found in jump performance.

**Conclusions:**

The school-based football programme, 11 for Health, with twice-weekly 45-min training sessions over 11 weeks improves various, but not all evaluated parameters related to musculoskeletal fitness in 10–12-yr-old Danish school children.

## 1 Background

Physical activity (PA) is now recognised as one of the most important factors contributing to a healthy lifestyle. Physical activity has positive impact on quality of life in childhood, adolescence, and adulthood, by reducing the effects of juvenile obesity associated to sedentary lifestyle currently considered a global public-health problem (Manzano-Carrasco et al., 2020; WHO, 2017). Following a structured Physical Activity (PA) program leads to a wide range of physiological and psychological benefits for adults, adolescents and children. Children and adolescents aged 6–19 years (generally defined as “youth”) are considered more physically active than adults in Denmark as well as elsewhere, but there are still room for improvements to fulfill the guidelines recommending at least 60 min d^−1^ of moderate-to-vigorous intensity PA (Andersen et al., 2016; Johansen et al., 2018; Department of Health and Human Services, 2019). Resistance training and activities leading to bone impact are strongly recommended on minimum 3 days per wk. (Department of Health and Human Services, 2019). In pre-adolescent children, PA is also useful for its psychological and social aspects (Silva et al., 2013) and team sports succeed in providing all these factors, from a healthy physical profile to social dynamics (Das and Horton, 2012). Football is widely known as the most popular and practiced sport in the world (Randers et al., 2010) with a workload that presents intermittent levels of intensity, and is further characterized by jumps, accelerations, sprints, changes of direction and running at different speed (Randers et al., 2014). Thus, football could elicit a better bone adaptation compared to long distance running conducted at constant intensity (Milanović et al., 2022). Indeed, during the sprint (Bangsbo, 1994; Freychat et al., 1996) rather strong ground reaction forces are activated which, combined with acceleration and deceleration movements, can stimulate the bone formation in the lower limbs and the axial skeleton. However, this osteogenic potential of running, is the subject of debate as lower bone mineral content (BMC) and density (BMD) have been found in male long-distance runners compared to inactive controls, whereas higher levels have been found among footballers (Calbet et al., 2001; Hetland et al., 1993).

Moreover, animal studies have shown that the osteogenic stimulus generated by mechanical impact depends on the degree of deformation of the bones induced by the external forces applied to them (Hart et al., 2017). Therefore, the greater the deformation rate and the more differentiated the distribution of deformation, the greater the osteogenic stimulus (Helge et al., 2014).

In accordance with this, cross-sectional studies conducted on athletes who practice weight-bearing and mechanical impact sports, which impose large and varied tensions on the skeleton, show a greater bone mass and BMD compared to counterparts not engaged in that type of exercise (Helge et al., 2014).

The peak bone mass that is build up during adolescence is probably a major determinant of risk of bone fracture later in life (Rizzoli et al., 2010). As it has previously been suggested, the peak bone mass can be raised by putting pressure on the bone tissue and therefore, sports that is characterized by impact on the ground, such as football, allow greater bone mass development compared to sports that do not have this characteristic, due to the work carried out by anti-gravity action (Vicente-Rodriguez et al., 2005; Vlachopoulos et al., 2017a). It has been speculated that after reaching an exercise-induced peak in bone mass, further increments due to the mechanical impact from the exercise would no longer be particularly high or tend to be less evident (Helge et al., 2014).

Furthermore, children aged 8–10 yrs who undergo sport activity protocols involving balls, have a higher aerobic and musculoskeletal score than children not involved in this type of exercise (Larsen et al., 2017a). This aligns with studies showing that children active in sports clubs with ball game activities are more likely to meet the guidelines regarding recommendations for daily physical activity, probably due to the high intensity of such activities compared to other types of physical activity (Hebert et al., 2015).

Recent studies on 8–10-year-old Danish children, shows that time spend in sports clubs contributes significantly to the amount of time children spend engaged in physical activity, including moderate to vigorous physical activity (Hebert et al., 2015). Therefore, such participation in ball games (including football) plays an important role in complying with the World Health Organization's standards for the 60 min of moderate-to-vigorous daily PA to be performed in order to obtain all the decanted and widely reported benefits upon health (Hebert et al., 2015; Nielsen et al., 2012; Bull et al., 2020). In fact, as many as 98 % of Danish boys and girls aged 9–11 who participate in 1–4 weekly training sessions with local football teams meet WHO recommendations regarding daily physical activity, with a corresponding 83 % for those participating in other sports club activities and 76 % for children that were not involved in any leisure-time sports club activities (Hebert et al., 2015).

Larsen et al., found that children who were active in sports clubs had higher total BMD compared to children not engaged in sports clubs. Total BMD was also higher in ball players than in non-active children in sports clubs (Larsen, 2016).

As for body composition, less data is available regarding young ball game players and children engaged in other sports clubs compared to age-matched children who do not participate in organized sports-club activities. There are, however, some indications that physical fitness and body composition are positively influenced by sports-participation, at least for some types of sport (Larsen et al., 2017, Larsen et al., 2021; Madsen et al., 2020a). These findings require confirmation in children from other countries and it would be of interest to study whether the body composition and musculoskeletal fitness components, such as muscle strength, postural balance and coordination, are linked to childrens' sports club participation (Larsen et al., 2017, Larsen et al., 2021; Madsen et al., 2020a). Studying Danish children who perform the “11 for Health” protocol may fill part of the research gap mentioned above and provide useful knowledge about the health effects of school based physical activity on leg and whole-body bone mineralization and muscular fitness in 10–12 year-old school children.

## 2 Methods

### 2.1 Design

**Fig. 1:**
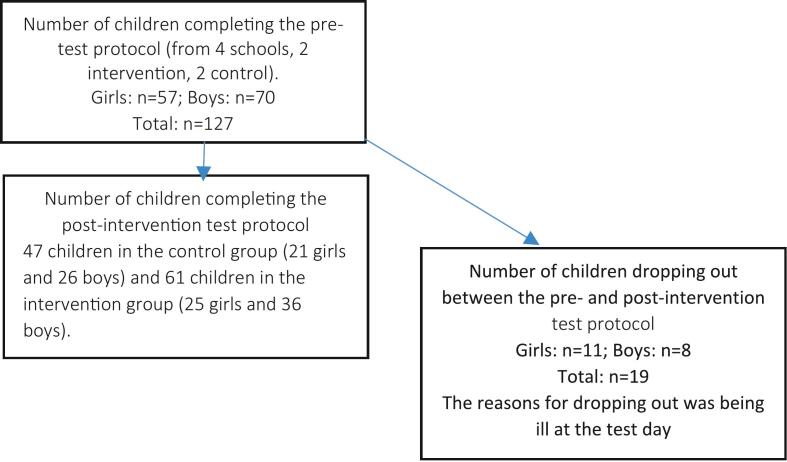
Study flow chart.

The study is a randomized trial using cluster randomization on school class level carried out in the Funen region (Denmark). All the participating schools agreed to implement the 11 for Health project (11fH), after invitation by e-mail. They signed up for 11fH before June 2019 and were invited for the present study in June by an additional e-mail. The schools came from one urban area and three rural cities which are considered as representable for all areas in Denmark (Fig. 1).

The study was conducted over three months for each class from August to December 2019 at the University of Southern Denmark in Odense. Parents/guardians gave their written and informed consents after full explanation of the aims, risks, benefits and procedures associated with the study before its beginning and were made aware that they could withdraw from the study at any time without further notice. The children were informed at the test day. The local ethics committee (ethic committee of Southern Denmark journal nr. H-16026885) approved this study design and procedures before the commencement of the study.

### 2.2 Measurements

Children's body composition was evaluated during a laboratory visit before and after the 11fH intervention. Measurements were done wearing light clothes with a whole-body Dual X-ray absorptiometry (DEXA) scan (Lunar Prodigy Advance, GE Medical Systems, Madison, Wisconsin, USA) with data collected using Encore software version 15 (Encore, Madison, WI, USA). The scanner was calibrated from the beginning of all testing days. DEXA variables used in the study were: Body Mass (BM), Lean body mass (LBM), Bone Mineral Content (BMC), Bone Mineral Density (BMD), and Fat Percentage (Fat%).

Height was measured prior the scan without footwear (Tanita, Tanita UK Ltd., Middlesex, UK). Standing Long Jump test (SLJ) and Stork balance test were employed to assess muscular skeletal fitness (Larsen et al., 2021a).

### 2.3 The intervention

The structure, content and implementation protocol for the “11 for Health in Denmark” programme has been described previously (Madsen et al., 2020b) and for this particular study it is important to highlight the involvement of physical activity with high intensity and muscular skeletal load (Ørntoft et al., 2017). The two weekly 45-minute sessions considered in the 11fH could be delivered within the curricular Physical Education class or during any other subject, provided they are separated by at least 40 h to optimise the physiological adaptations. The programme began with a 20-hour (spread over 3 continuous days) interactive training course for teachers from each of the participating school. The courses were arranged by the Danish Football Association (DBU) and the University of Southern Denmark (SDU), and taught by experienced football coaches and scientists, who demonstrated the football skills and health-related discussions as well as explaining the tests included in the project. At the end, the ability of the participating teachers and educators to deliver the sessions was tested using a series of teach-backs. All course participants received a comprehensive manual describing the philosophy of the programme and the potential effects of the programme on well-being and health profile. The manual also outlines the skills required to teach the programme in an enjoyable yet educational way and define the content and timing of each feature of the programme. Finally, the manual contains information on the relevant health knowledge associated with each session.

### 2.4 Statistical analysis

Statistical analyses were performed using Sigmaplot 14.0 (Chicago, Illinois), significance was accepted at *p* < 0.05. Descriptive statistics for height, weight and age are presented in Table 1 and data are expressed as mean ± *SD*.

**Table 1:** Subject characteristics.

	CG *(n* *=* *47)*		IG *(n* *=* *61)*	
	*Pre*	*Post*	*Pre*	*Post*
Age (y)	10.7 ± 0.5	11.0 ± 0.3	10.7 ± 0.5	11.0 ± 0.4
Height (cm)	149.5 ± 8.4	151.2 ± 8.8	148.1 ± 6.1	150.0 ± 6.5
Weight (kg)	40.9 ± 8.3	42.2 ± 8.6	41.3 ± 10.0	43.5 ± 10.8

Data are presented as means ± SD.

Delta values (Δ) were calculated between pre and post measurements for the control group (CG) and the intervention group (IG). A mixed ANOVA design was used to analyze between-group and within-group differences.

## 3 Results

### 3.1 Sample population

A total of 108 children aged 10–12 participated in the study, with 47 children in the control group (21 girls and 26 boys) and 61 children in the intervention group (25 girls and 36 boys) assigned randomly. At baseline, there were no significant differences in the age, height, weight (Table 1) fat percentage, lean mass (Table 2) between IG and CG (*p* > 0.05).

**Table 2:** Body composition before (Pre) and post (Post) the 11-week study period for the CG and IG included into the two weekly 45-min 11 for Health sessions.

	C (n = 47)		I (n = 61)		Δ		DiD
	*Pre*	*Post*	*Pre*	*Post*	*CG*	*IG*	*IG vs CG*
Leg BMC (g)	559.43 ± 109.80	582.74 ± 112.51	551.94 ± 99.40	575.15 ± 102.52	23.32 ± 10.49	23.21 ± 12.91	−0.11
TBLH BMC (g)	1143.85 ± 208.79	1192.94 ± 217.91	1133.09 ± 186	1175.98 ± 195.11	49.09 ± 24.64	42.89 ± 26.46	−6,20
Total mass (kg)	41.59 ± 8.23	42.26 ± 8.59	42.27 ± 10.39	43.52 ± 10.73	1.20 ± 0.94	1.24 ± 0.87	0.04
Leg BMD (g/cm^2^)	0.912 ± 0.091	0.926 ± 0.089	0.889 ± 0.079	0.917 ± 0.084	0.014 ± 0.018	0.021 ± 0.019	0.007^a^
TBLH BMD (g/cm^2^)	0.7950 ± 0.080	0.808 ± 0.075	0.787 ± 0.071	0.801 ± 0.067	0.012 ± 0.015	0.014 ± 0.015	0.002
Leg LBM (kg)	9.46 ± 1.81	9.77 ± 1.89	9.29 ± 1.77	9.80 ± 1.94	0.32 ± 0.35	0.51 ± 0.46	0.20^a^
Total LBM (kg)	28.29 ± 4.59	29.04 ± 4.82	27.79 ± 4.32	28.89 ± 4.60	0.75 ± 0.72	1.10 ± 0.83	0.35
Total Fat %	26.5 ± 7.5	26.6 ± 7.8	29.0 ± 8.0	28.5 ± 8.0	0.1 ± 0.1	−0.6 ± 0.1	−0.7^a^

Data are presented as means ± SD.

a Significant between-group difference; C = Control Group; I = Intervention Group; Δ = Delta (post – pre); DiD = Differences in Delta values; TBLH = Total Body Less Head; BMD = Bone Mineral Density; BMC = Bone Mineral Content; LBM = Lean Body Mass.

### 3.2 Body composition

During the 11-week study period, leg BMD as well as leg LBM increased more in the IG compared to CG (0.021 ± 0.019 *vs* 0.014 ± 0.018 g/cm^2^, 0.51 ± 0.46 *vs* 0.32 ± 0.35 kg, p < 0.05) whereas the body fat percentage decreased more in IG than in CG (−0.6 ± 0.1 *vs* 0.1 ± 0.1 %-points, *p* < 0.001). No differences between delta values were found in the age, height, weight and BMC. All results from the DXA-scanner are reported in (Table 2).

### 3.3 Muscular skeletal fitness

After the 11-week period, between-group differences were observed in favor of IG compared to CG. Stork balance test performance increased more in IG than in CG (0.5 ± 2.6 *vs* − 1.5 ± 4.4 s, *p* < 0.05). Moreover, IG tended to have a greater improvement in SLJ test performance during the 11-week period compared to CG (3.6 ± 10.2 *vs* 0.2 ± 9.8 cm, *p* = 0.08). All results related to muscular skeletal fitness are presented in Table 3.

**Table 3:** Muscular Skeletal Fitness Test results before (Pre) and post (Post) the 11-week study period for the CG and IG including into the two weekly 45 min. 11 for Health sessions.

*Test*	C		I		Δ		DiD
	*Pre*	*Post*	*Pre*	*Post*	*CG*	*IG*	*IG vs CG*
SLJ (cm)	126.9 ± 20.1	127.3 ± 19.6	117.2 ± 18.6	120.0 ± 18.6	0.2 ± 9.8	3.6 ± 10.2	3.4
Postural balance (s)	5.7 ± 3.9	4.2 ± 2.2	3.8 ± 1.9	4.4 ± 2.3	−1.5 ± 4.4	0.5 ± 2.6	2.0^a^

Data are presented as means ± SD.

a Significant between-group difference; C = Control Group; I = Intervention Group; Δ = Delta (post – pre); DiD = Differences in Delta values; SLJ = Standing Long Jump.

## 4 Discussion

The main findings from the present study were that the 11 for Health program, with twice-weekly 45-min training sessions over 11 weeks, results in an improvement on leg bone mineralization and muscle mass for 10–12-year-old Danish school children, thereby confirming that small-sided football training provides significant osteogenic stimuli and muscular impact, especially for the lower body.

The observed between-group difference of 7 mg/cm^2^ in leg bone mineral density was smaller than observed in another sports-based school intervention study in Danish school children aged 8 to 10 years, but it should be emphasized that higher training volumes and training duration were used in previous study (Larsen et al., 2018). In the study by Larsen et al. (Larsen et al., 2018) an increase of 19 mg/cm^2^ (change score compared to control) after 10 months of three 40-min sessions with small-sided ball games per week was described, whereas 12-min sessions on all schooldays did not result in a significant increase (Larsen et al., 2017b). Together these findings confirm that football training with high bone impact (Ørntoft et al., 2017), can positively affect bone health in children as long as the duration of each session are not too short. Moreover, the present study revealed increases in leg muscle mass and postural balance and a tendency for an elevated standing long jump performance. These findings are in line with the observation of a positive training-induced effect on leg bone mineralization, as the region specific lean mass is suggested to be the strongest determinant of BMD (Vlachopoulos et al., 2017b). The present study also indicate some positive effects on body fat percentage, with absolute changes that are in the same range as observed in previous *11 for Health* studies using bioimpedance (Ørntoft et al., 2016), all together supporting a positive impact on body composition and the general health profile (Cummings et al., 1993; Berenson, 2002). Youngsters are more physiologically adaptive to stimuli resulting from aerobic exercise training (Rowland, 2013), resistance training (Myers et al., 2017), and bone leading exercise (Specker et al., 2015; Francis et al., 2014). Indeed, pre-pubertal children have achieved strength gains similar to those engaged in resistance training registered in adolescents (Malina, 2006). Moreover, evidence suggests that aerobic- and resistance exercise training lead to improvements in body composition, bone strength and benefits cardiovascular disease risk factors and prevention of sports-related injuries (Department of Health and Human Services, 2019), whereas more studies investigating the effects of recreational football on bone health is needed, since there was only few studies with children in recent reviews covering this.

The beneficial role of PA on bone health particularly is widely and strongly supported in literature. A review consisting of 22 intervention trials in children and youth aged 3–18 years underlined an annual increase in bone accrual of 0.6–1.7 % due to PA or an exercise intervention (Specker et al., 2015). However, we are still not able to clearly identify and establish the precise FITT (Frequency-Intensity-Time-Type) prescription in order to improve bone health (Weaver et al., 2016). Some important factors, which produce an increase in bone health as outcome, should be considered in children and adolescent training programs. Bones respond to PA or stressing stimuli which produce a strain on bones themselves due to the impact or to the mechanical load. Moreover, the response is much higher as stimuli are dynamic, short in time, with moderate-to-high intensity, but not affected by the direction of the applied load (Weaver et al., 2016; Liguori and Medicine, 2020).

The “*11 for Health*” program demonstrates positive effects on body composition and bone health as described above and has proved to be implementable in large scale and has the potential to involve all children due to the popularity of the program (Larsen et al., 2021b). The applied approach with a cluster randomization design is one of the study's strengths along with golden standard measures provided by the DEXA scanner, whereas the limited number of participants can be seen as a limitation, as both genders are analysed together and by the lack of measures of potential confounding factors like puberty, calcium intake, vitamin D, physical activity levels. Also, the time between the measures is low, only 3 months compared to typically 6 months or more. This leads to small detectable changes just around the limit for expected precision of the scanner (Leonard et al., 2009), and the results must be interpretated with that in mind. The findings related to fat percentage, postural balance and standing long jump performance must also be interpretated in the light of the baseline performance, where the intervention group had lower scores, and thereby a potential better chance for enhancements. Moreover, the training intensity and musculoskeletal impact during the training sessions were not obtained from the present study, but from previous studies with other subjects using the same protocol (Ørntoft et al., 2017).

## 5 Conclusions

The 11-week school-based “*11 for Health*” programme with health education through football seems to improve body composition and muscular skeletal health for 10–12-yr-old school children. Body fat percentage as well as leg BMD and LBM was improved compared to control after the intervention and so was the balance test performance.

## CRediT authorship contribution statement

MNL and AT performed the statistical analysis, conducted testing, analysed the data, prepared the first draft of the paper, revised the manuscript, and approved the final version of the paper. CC and PB contributed to the design of the study, revised the manuscript and approved the final submission. TKM and CSA implemented the intervention, conducted testing, analysed the data, revised the manuscript and approved the final submission. PK designed the study, applied for funding, implemented the intervention, revised the manuscript and approved the final version of the paper.

## Funding

The 10.13039/501100004825Nordea Foundation (Nordea-fonden), the 10.13039/501100012352Danish Football Association (DBU) and Aase and Ejnar Danielsens Foundation.

## Declaration of competing interest

The authors declare that they have no known competing financial interests or personal relationships that could have appeared to influence the work reported in this paper.

## Data Availability

The authors do not have permission to share data.
